# Prevalence and Impact of Sleep Disordered Breathing in Patients with Severe Aortic Stenosis

**DOI:** 10.1371/journal.pone.0133176

**Published:** 2015-07-27

**Authors:** Markus Linhart, Jan-Malte Sinning, Alexander Ghanem, Finny J. Kozhuppakalam, Rebecca Fistéra, Christoph Hammerstingl, Carmen Pizarro, Eberhard Grube, Nikos Werner, Georg Nickenig, Dirk Skowasch

**Affiliations:** Medizinische Klinik und Poliklinik II, Universitätsklinik Bonn, Sigmund-Freud-Str. 25, 53127 Bonn, Germany; KRH Robert Koch Klinikum Gehrden, GERMANY

## Abstract

**Background:**

Unlike the well-established association between sleep disordered breathing (SDB) and chronic heart failure, the relationship between SDB and severe aortic stenosis (AS) is not well investigated. Given the increasing prevalence of AS, and the improving prognosis of high risk AS patients attributable to transcatheter aortic valve implantation (TAVI), the prevalence and impact of SDB needs to be better understood.

**Methods and Results:**

In this study, 140 patients with severe AS underwent polygraphy prior to TAVI. Clinical and hemodynamic parameters were recorded. Patients were followed for 573±405 days. We found that 99/140 patients (71%) had SDB with a mean apnoea-hypopnoea-index of 24±17/h. SDB was mild in 27%, moderate in 23% and severe in 21% of patients. In addition, 35 patients (25%) had obstructive sleep apnoea (OSA), whereas 64 patients (46%) had central sleep apnoea (CSA). Patients with OSA had predominantly mild SDB (20/38 pts.), and patients with CSA mostly had severe SDB (24/29 pts.). The prevalence and distribution of OSA and CSA were independent of left ventricular function. Overall, 1 and 2 year survival rates (74% and 71%, resp.) did not differ significantly between patients without SDB or those with OSA and CSA (p=0.81).

**Conclusions:**

SDB, with a preponderance of CSA, was found to be highly prevalent in patients with high-grade AS scheduled for TAVI. SDB prevalence was independent of left ventricular function. Mortality after TAVI was not influenced by the type or severity of SDB.

## Introduction

There are many associations between cardiovascular diseases and sleep disordered breathing (SDB). In particular, the obstructive form of SDB (obstructive sleep apnoea, OSA) has been identified as an important risk factor for a variety of cardiovascular diseases such as arterial hypertension, coronary artery disease and atrial fibrillation [1]. On the other hand, central sleep apnoea (CSA) has mainly been associated with chronic congestive heart failure (CHF) and carries a significantly adverse prognosis [2]. Studies have shown that treatment of the associated cardiac condition can improve the underlying sleep disorder, and vice versa [3–6]. However, preliminary data from the recent SERVE-HF trial showed an increase in cardiovascular mortality in patients with heart failure with reduced ejection fraction and predominant CSA that were treated by adaptive servo-ventilation (http://www.servehf.com) [7]. To date, scarce data exist about the relationship between valvular heart disease and SDB [8–10]. Aortic stenosis is the most frequent valvular heart disease and its prevalence is expected to increase due to aging populations [11]. The aim of this study was to investigate the frequency of SDB in patients with severe aortic stenosis and its impact on mortality after transcatheter aortic valve implantation (TAVI). We found that patients with severe aortic stenosis scheduled for TAVI had a high prevalence of SDB with a preponderance of CSA and that this was not associated with outcomes after TAVI.

## Methods

### Study Design and Patient Selection

A total of 140 patients with high-grade aortic stenosis who were evaluated for TAVI at our centre between 2010 and 2013 were screened prospectively for our study using polygraphy. The study was approved by the local medical ethics committee (Ethikkommission an der Medizinischen Fakultät der Rheinischen Friedrich-Wilhelms-Universität Bonn, project approval no. 255/08). Informed written consent was obtained from all patients. All patients underwent our standard preoperative diagnostic tests, which included comprehensive 2D- and 3D-transthoracic and transesophageal echocardiography (Philips iE 33 ultrasound system, Amsterdam, The Netherlands) as well as coronary angiography with invasive determination of left ventricular end-diastolic pressure (LVEDP) [12, 13]. Left ventricular end-diastolic volume, left ventricular end-systolic volume, left ventricular ejection fraction (LVEF) and left atrial volume were determined by 2D echocardiography. Right ventricular systolic pressure was estimated on the basis of the modified Bernoulli equation and was considered equal to the systolic pulmonary artery pressure (sPAP). Body surface area was calculated using the DuBois formula. The 30-day mortality risk was estimated by means of the logistic EuroSCORE (www.euroscore.org) and the Society of Thoracic Surgeons (STS) score ((http://riskcalc.sts.org/) algorithms that are based on the presence of coexisting illnesses.[14] Patients were followed on an outpatient basis 180 and 365 days after the procedure and every year thereafter. Patients who did not come to their follow-up appointments were interviewed by telephone.

### Sleep Study and Scoring

Sleep studies were performed by ambulatory polygraphy as described previously [10]. The studies were performed by unattended in-hospital 6-channel polygraphy during the screening period prior to TAVI. An Embletta polygraph (Embla Ltd., Amsterdam, The Netherlands), which records nasal pressure and airflow, chest and abdominal wall movements, oxygen saturation, and heart rate continuously, was used for all recordings. Episodes of disordered breathing were categorised as apnoea or hypopnoea, and as either obstructive or central. Apnoea was defined as a cessation of airflow or a >90% reduction in airflow from baseline for >10 seconds. Hypopnoea was defined as a reduction in airflow of >30% with an oxygen desaturation of ≥4%. The apnoea–hypopnoea index (AHI) was defined as the number of episodes of apnoea and hypopnoea per hour. Patients with an AHI >5/h were considered to have SDB [15], which was subclassified into mild, moderate, and severe forms with 5–14.9, 15–29.9, and ≥30 events/hour, respectively. Events were scored manually by an experienced sleep laboratory specialist. Events were classified as obstructive when the airflow criteria were met and thoracic or abdominal respiratory effort was documented; otherwise they were classified as central. An episode of SDB in which more than 50% of the events were central was defined as CSA whereas those in which ≥ 50% of the events were obstructive was defined as OSA. Mixed apnoea was defined as an absence of nasal airflow associated with no respiratory effort followed by the resumption of inspiratory effort in the second portion of the event. These events were classified as part of the OSA group [16]. The central apnoea index (CAI) and the obstructive apnoea index (OAI) were calculated as the mean number of episodes of central and obstructive apnoea events per hour, respectively. The oxygen desaturation index (ODI) was defined as the number of oxygen level drops of ≥3% from baseline per hour. The hypopnoea index (HI) was defined as the number of hypopnoeas per hour. Sleep-related symptoms were documented with the German version of the self-rating Epworth Sleepiness Scale questionnaire (ESS) [17]. A result in the 0–9 range was considered normal whereas a result in the 10–24 range indicated daytime sleepiness [18].

### Statistical Analysis

Continuous variables are expressed as the mean ± one standard deviation and were analysed by a two-sided unpaired Student’s *t*-test. For values of N-terminal pro–B-type natriuretic peptide with a highly skewed distribution, statistical significance was calculated using the Wilcoxon–Mann–Whitney test. Categorical variables were compared by Fisher’s exact test or the chi-square test for trend, as appropriate. Correlation between measures was calculated using Spearman’s rank correlation. Survival rates were compared with the log-rank and Breslow test. Statistical analyses were performed with SPSS software version 22.0 (IBM Inc., Armonk, New York, USA). A p value <0.05 was considered statistically significant.

## Results

### Baseline characteristics

140 patients were included in the study. The mean age of the patients was 81±6 years, and 52% of the patients were male. The aortic valve areas were narrowed to an average of 0.39±0.11 cm^2^/m^2^ with a mean peak-to-peak gradient of 47±24 mmHg. Mean EuroSCORE was 23±16%. Patients were not obese, with a mean BMI of 26±4 kg/m^2^. On average, renal function was only mildly impaired (eGFR 55±22 ml/min) and mean left ventricular systolic function was only mildly reduced (54±17%). Table 1 displays the clinical and hemodynamic parameters of the patients at baseline.

**Table 1:** Baseline patient characteristics.

	n = 140
Age (years)	81.4 ± 6.2
Male sex, n (%)	73 (52%)
NYHA	3.1 ± 0.5
ESS	7.8 ± 4.3
EuroSCORE	22.6 ±15.8
STS risk score	9.4 ± 7.0
Coronary artery disease, n (%)	89 (64%)
Atrial fibrillation, n (%)	53 (38%)
Previous stroke, n (%)	18 (13%)
BMI (kg/m^2^)	25.8 ± 4.3
eGFR (ml/min)	55 ± 22
NT pro-BNP (pg/ml)	5763 ± 7704
Aortic valve area index (cm^2^/m^2^)	0.39 ± 0.11
Peak-to-peak gradient (mmHg)	47 ± 24
LVEDP (mmHg)	20 ± 9
LVEF (%)	54 ± 17
sPAP (mmHg)	38 ± 16
Left atrial volume index (mL/m^2^)	55 ± 31

ESS Epworth sleepiness scale, STS Society of Thoracic Surgeons, BMI body mass index, LVEF left ventricular ejection fraction; LVEDP, left ventricular end-diastolic pressure, sPAP systolic pulmonary artery pressure.

### Prevalence of sleep-disordered breathing

**Fig 1:**
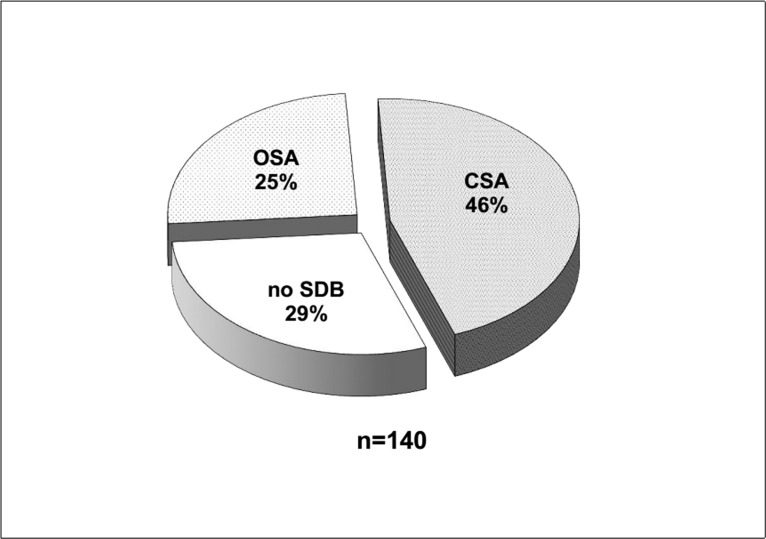
Prevalence and type of sleep disordered breathing (SDB) in 140 patients with severe aortic stenosis.

**Fig 2:**
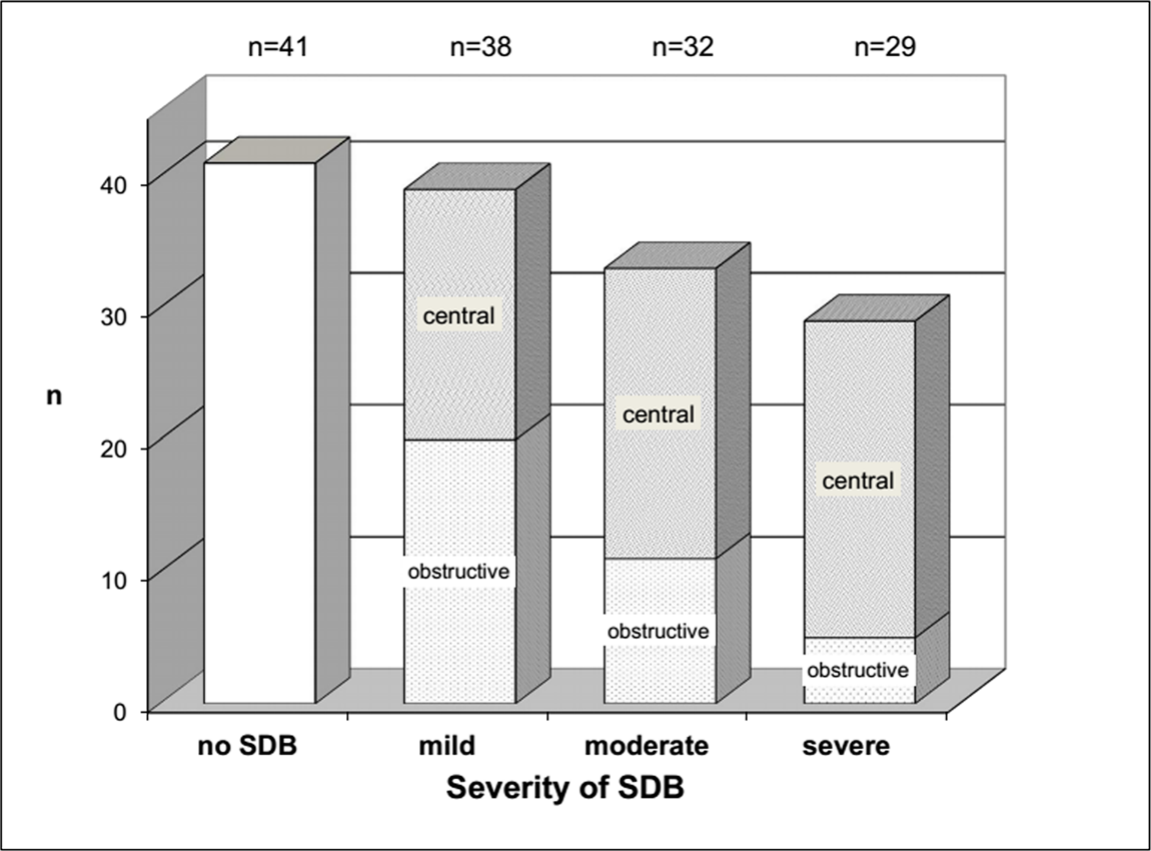
Distribution of sleep disordered breathing. With increasing severity of sleep disordered breathing (SDB), the prevalence of the central subtype increases at the expense of the obstructive subtype of SDB. Severity is indicated by the apnoea-hypopnoea-index: mild 5–14.9/h, moderate 15–29.9/h, severe >30/h.

The prevalence of SDB as defined by an AHI >5/h was 71% (99/140 pts.). Mean AHI in patients with SDB was 24.0±17.2/h. Fig 1 shows the distribution of SDB types. One-third of the patients with SDB (35 pts., 35%) displayed an obstructive sleep apnoea pattern, whereas two-thirds (64 pts., 65%) displayed a central sleep apnoea pattern. When stratified by the severity of SDB as defined by AHI, there was a growing preponderance of CSA at the expense of OSA with increasing SDB severity. The ratio of CSA to OSA in mild SDB was approximately 1:1 whereas it reached almost 5:1 in severe SDB (Fig 2).

### Relationship between SDB and LVEF as well as other clinical and hemodynamic parameters

**Fig 3:**
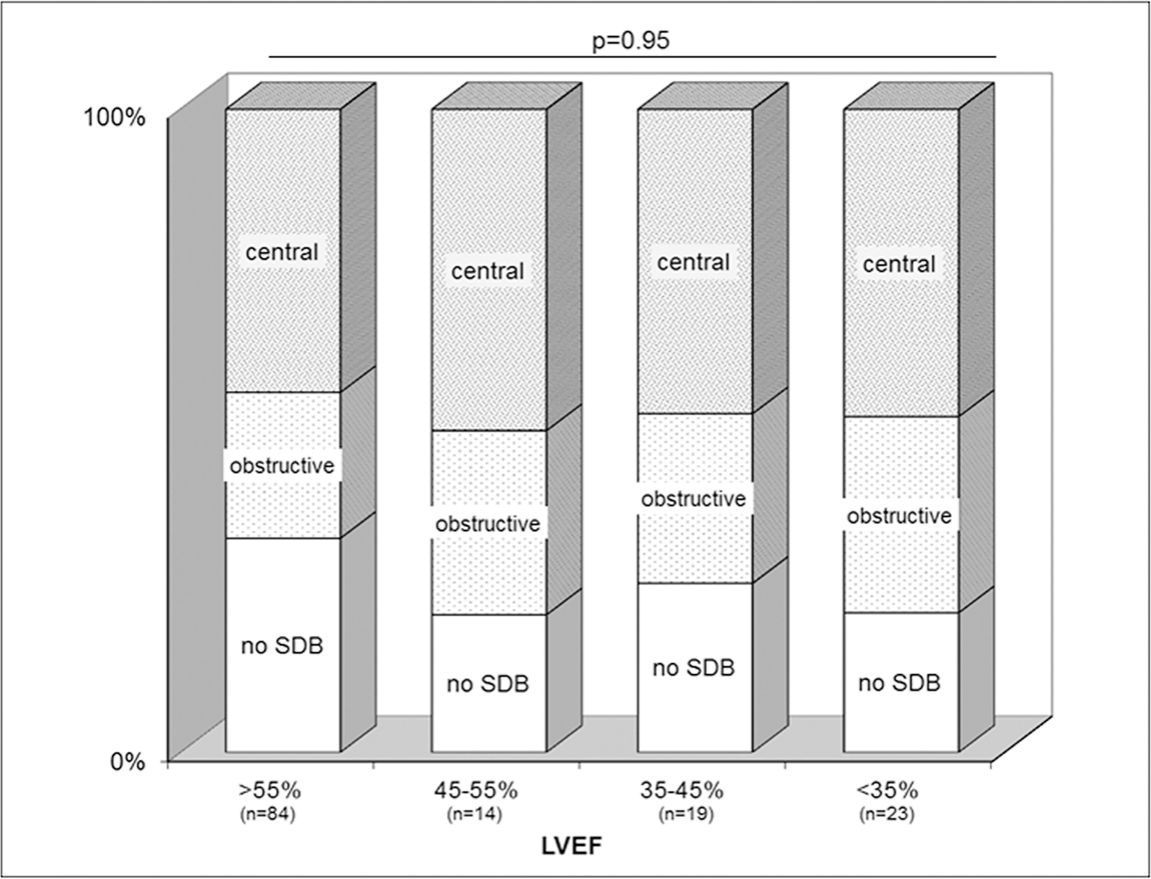
Distribution of sleep apnoea, and its central and obstructive subtypes, is independent of left ventricular ejection fraction.

The prevalence of SDB and distribution of its subtypes (OSA and CSA) was independent of the left ventricular ejection fraction. Mean LVEF was not different between the groups (Table 2). When stratified into categories of normal, mildly reduced, moderately reduced and severely reduced LVEF, there was a similar distribution of SDB in each category with no statistically significant differences (Chi-square test for independence, p = 0.95, Fig 3). Likewise, LVEDP and NT pro-BNP levels were not significantly different between the groups (Table 2). Although renal dysfunction may have been a confounder for the raised NT pro-BNP levels, our findings remained consistent even when patients with renal dysfunction was excluded from the analysis (p = 0.56).

**Table 2:** Comparison of clinical and hemodynamic parameters between patients without SDB or with OSA or CSA.

Parameter	No SDB	OSA	CSA			
	n = 41	n = 35	n = 64	p-value		
				no SDB vs. OSA	no SDB vs. CSA	OSA vs. CSA
Age (years)	79.4 ± 7.3	83.4 ± 4.8	81.6 ± 5.8	0.005	0.11	0.12
Male sex, n (%)	19 (46%)	11 (31%)	43 (67%)	0.24	0.043	0.001
NYHA	3.1 ± 0.5	3.2 0.4	3.1 ± 0.5	0.32	0.89	0.20
ESS	6.7 ± 2.7	9.3 4.1	7.6 ± 4.9	0.008	0.39	0.11
EuroSCORE	18.0 ± 13.6	22.5 ± 12.6	25.5 ± 18.2	0.16	0.027	0.37
STS risk score	8.0 ± 6.8	9.7 ± 5.4	10.0 ± 7.9	0.29	0.24	0.87
Coronary artery disease, n (%)	22 (54%)	23 (66%)	44 (69%)	0.35	0.15	0.82
Atrial fibrillation, n (%)	15 (38%)	14 (40%)	24 (38%)	1.00	1.00	0.83
Previous stroke, n (%)	7 (17%)	3 (9%)	8 (13%)	0.33	0.57	0.74
BMI (kg/m2)	25.3 ± 3.5	26.8 ± 4.5	25.5 ± 4.6	0.11	0.77	0.19
eGFR (ml/min)	58 ± 22	55 ± 22	53 ± 21	0.60	0.25	0.61
NT pro-BNP (pg/ml)	4542 ± 4995	6457 ± 10350	6111 ± 7380	0.19	0.83	0.45
Aortic valve area index (cm2/m2)	0.39 ± 0.09	0.38 ± 0.11	0.39 ± 0.11	0.61	0.81	0.78
Peak-to-peak gradient (mmHg)	44 ± 21	44 ± 24	50 ± 25	0.92	0.28	0.26
LVEDP (mmHg)	22 ± 10	20 ± 8	19 ± 8	0.28	0.09	0.65
LVEF (%)	55 ± 16	52 ± 16	53 ± 17	0.38	0.61	0.65
sPAP (mmHg)	38 ± 18	35 ± 12	41 ± 17	0.39	0.59	0.10
Left atrial volume index (mL/m2)	54 ± 29	56 ± 19	55 ± 37	0.80	0.94	0.89
AHI (1/h)	1.9 ± 1.6	18.4 ± 15.2	27.5 ± 17.6	<0.0001	<0.0001	0.011
ODI (1/h)	3.8 ± 9.5	15.0 ± 16.1	21.5 ± 18.1	<0.0001	<0.0001	0.081
HI (1/h)	1.0 ± 1.2	6.6 ± 6.6	6.1 ± 6.2	<0.0001	<0.0001	0.735
AI obstructive (1/h)	0.4 ± 0.7	9.5 ± 11.6	2.9 ± 4.9	<0.0001	<0.0001	0.003
AI central (1/h)	0.5 ± 0.9	1.8 ± 2.5	16.5 ± 14.0	0.004	<0.0001	<0.0001

ESS Epworth sleepiness scale, STS Society of Thoracic Surgeons, BMI body mass index, eGFR estimated glomerular filtration rate, LVEF left ventricular ejection fraction, LVEDP left ventricular end-diastolic pressure, sPAP systolic pulmonary artery pressure, AHI apnea-hypopnea-index, ODI oxygen desaturation index, HI hypopnoea index, AI apnoea index.

There was a weak but significant positive correlation between AHI and left atrial volume index (r = 0.31, p = 0.02) in the CSA but not the OSA group. No significant correlations were seen between SDB, in terms of the overall group as well as the OSA and CSA subgroups, and multiple clinical parameters including LVEF, LVEDP, NT pro-BNP and sPAP.

### Relation of SDB to outcome after TAVI

**Fig 4:**
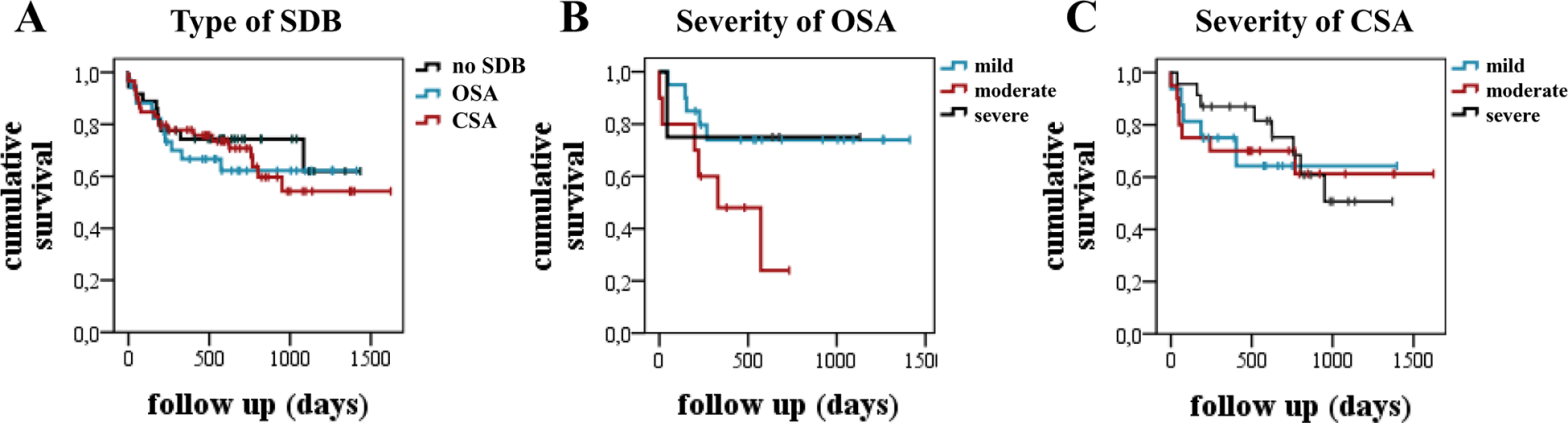
Kaplan-Meier estimate of survival. Kaplan-Meier estimates of survival with regard to the type of sleep disordered breathing (SDB, Panel A) and the severity of obstructive (OSA, Panel B) and central sleep apnoea (CSA, Panel C), respectively, show no significant differences in mortality between the groups. Mild SDB: AHI 5–14.9/h, moderate SDB: AHI 15–29.9/h, severe SDB AHI >30/h.

Median patient follow-up was 573±405 days. A total of 11 patients were lost to follow-up. Overall, one and two year survival rates were 74.4% and 71.3%, respectively. The rates of survival were not significantly different in patients without SDB and those with OSA or CSA (p = 0.81, Fig 4A, Table 3A and 3B). There was also no significant difference observed in the survival rates of patients with OSA or CSA with regard to the severity of SDB (p = 0.14 and p = 0.88, resp., Fig 4B and 4C, Table 3C–3F).

**Table 3:** Statistics of survival analysis.

A Type of SDB: mean of survival time (days) (Fig 4A)				
	estimate	standard error	95%-confidence interval	
			lower	upper
no SDB	1.058.291	100.162	861.973	1.254.609
OSA	955.134	105.758	747.849	1.162.419
CSA	1.075.131	96.788	885.426	1.264.837
total	1.104.801	65.020	977.362	1.232.239
B Type of SDB: Comparison of survival rates with log rank and Breslow test				
	test	chi-square	df	significance
	log rank	0.427	2	0.808
	Breslow	0.460	2	0.794
C Severity of OSA: mean of survival time (days) (Fig 4B)				
	estimate	standard error	95%-confidence interval	
			lower	upper
mild	1.089.531	124.649	845.219	1.333.843
moderate	396.400	88.570	222.804	569.996
severe	860.000	235.559	398.305	1.321.695
total	955.134	105.758	747.849	1.162.419
D Type of SDB: Comparison of survival rates with log rank and Breslow test				
	test	chi-square	df	significance
	log rank	3.995	2	0.136
	Breslow	3.026	2	0.220
E Severity of CSA: mean of survival time (days) (Fig 4C)				
	estimate	standard error	95%-confidence interval	
			lower	upper
mild	962.438	161.092	646.697	1.278.178
moderate	1.083.738	163.706	762.874	1.404.601
severe	986.950	104.539	782.054	1.191.847
total	1.075.131	96.788	885.426	1.264.837
3F Type of SDB: Comparison of survival rates with log rank and Breslow test				
	test	chi-square	df	significance
	log rank	0.253	2	0.881
	Breslow	1.051	2	0.591

## Discussion

This is the largest study to date investigating SDB in patients with severe aortic stenosis.

The main findings are:

1. There is a high prevalence of SDB (71%) in this patient population, with a preponderance of the CSA subtype (46%).
2. With increasing severity of SDB as defined by AHI, the prevalence of CSA increases at the expense of OSA.
3. The prevalence and distribution of SDB is independent of the left ventricular function and not associated with survival after TAVI.

Unlike in CHF, arterial hypertension or atrial fibrillation, the association between valvular heart disease and SDB is not well characterised. Aortic stenosis is the most common valvular heart disease in developed countries and its impact on public health and health care resources is expected to grow due to aging populations [11]. Concurrently, because of the development of TAVI, an increasing number of AS patients with high or excessive surgical risk can be effectively treated. TAVI can therefore greatly improve life expectancy in these patients [19]. With ongoing technical improvements, TAVI is expected to become a good alternative to surgery for an increasingly broader range of patients [19]. Therefore, the significance of SDB in these patients needs to be evaluated. Such studies would also allow for greater insights into the mechanism of SDB in cardiac disease.

### Prevalence of SDB

We found that there is a very high prevalence of SDB in the AS patient population, with an overall prevalence of 71%. This finding confirms a previous, smaller report, where SDB was found in 30/42 patients with severe aortic stenosis [9]. The prevalence of SDB in AS patients is thus similar to the prevalence in SDB in CHF patients as described in previous studies. For instance, a study of 700 patients with CHF, as defined by NYHA class ≥II and LVEF ≤40%, found similar rates of SDB as our study both in terms of the overall prevalence rate as well as the distribution of CSA and OSA [20]. In our study, CSA accounts for the majority of moderate and severe cases of SDB (46/61 pts., 75%) whereas OSA prevails in the mild forms (20/38 pts., 53%). By nature, patients eligible for TAVI are of advanced age. In our study cohort, mean age was 81±6 years. It has been suggested that SDB is age-dependent in elderly individuals whereas it seems to be age-related in middle age individuals [21, 22]. In a recent study in 298 elderly women with a mean age of 82±3 years, 35% had an AHI >15/h, as compared with 44% of patients in our study [23]. Taking together the available data, SDB seems to be highly prevalent also in the general elderly population. However, definition and diagnosis of SDB and its subtypes were varying in the studies, and details on cardiovascular comorbidities were not reported. Thus, to date it remains unclear to what proportion of SDB is age-dependent and what is attributable to severe cardiac disease such as CHF or severe aortic stenosis in this population. Our findings demonstrate that SDB, in particular CSA, constitutes a frequent comorbidity in patients with severe aortic stenosis and requires further investigation as part of the clinical workup.

### SDB and left ventricular function

Interestingly, the proportion of SDB and its subtypes was found to be independent of left ventricular systolic and diastolic function. Mean LVEF was not different between the groups, nor was the distribution of SDB when stratified according to the degree of LVEF impairment (Table 2, Fig 3). SDB was also present in a considerable number of patients with normal LVEF. Likewise, NT pro-BNP was strongly elevated in all groups but was not significantly different between the groups (Table 2). This finding held true even if the analysis was limited to only patients with normal or mildly reduced renal function. This is in contrast to a study of chronic heart failure without valvular disease, where men with low levels of the natriuretic peptide BNP were found to have a low risk of CSA [24]. These findings implicate that SDB in severe AS, as opposed to SDB in CHF, is not directly related to systolic heart failure.

### Pathophysiology of SDB in cardiac disease

The mechanistic link between SDB and cardiac disease is not yet fully understood. OSA is believed to be a risk factor for many cardiac diseases by inducing increases in afterload as well as elevations in systemic blood pressure secondary to hypoxia and increasing sympathetic nervous system activity during sleep disordered breathing events. These factors ultimately exert harmful effects on the cardiovascular system [1]. Such a mechanism may also be the underlying cause of the development of calcific aortic stenosis, which shares the same risk factors with coronary artery disease [25, 26]. The current theory of cardiovascular pathogenesis in CSA, in brief, involves pulmonary congestion secondary to heart failure that leads to chronic hyperventilation from repeated pulmonary vagal irritant receptor stimulation. This, in turn, facilitated by enhanced chemosensitivity to CO_2_, drives the PaCO_2_ below the apnoea threshold, and triggers episodes of apnoea [27]. Prior studies in patients with CSA have shown a positive correlation between pulmonary capillary wedge pressure and the frequency and severity of central apnoea [16]. In our study cohort, pulmonary capillary wedge pressure was not systematically evaluated. We were unable to document a significant correlation between clinical parameters that, within certain limitations, could indicate an increased pressure load in the pulmonary circulation (such as echocardiographically estimated systolic pulmonary pressure (sPAP) or invasively determined LVEDP). However, we found a weak but significant, positive correlation between AHI and left atrial volume in the CSA group. This suggests that in left atrial dilatation due to left atrial stretch might be involved in the underlying pathophysiology of CSA.

The recent fluid shift hypothesis suggests that nocturnal fluid shifts contribute to the pathogenesis of both OSA and CSA in patients with CHF. This is thought to occur by means of fluid displacement from the legs to the neck during nocturnal recumbency, which causes an obstruction of the upper airways and, in CSA, also to the lungs, where it aggravates the pulmonary congestion [28]. It is conceivable that this mechanism also applies to SDB in severe aortic stenosis; however, specific measurements such as changes in leg fluid volume and neck or calf circumference [28] were not evaluated in our study. Moreover, routine hemodynamic parameters such as those discussed above might not be sensitive enough to predict SDB and its subtypes in this patient group.

### Outcomes after TAVI and clinical perspective

Our findings show that SDB is a very frequent comorbidity in patients with severe aortic stenosis who are eligible for TAVI. We studied a very particular patient population that had malignant valvular disease, advanced age, many comorbidities and significant frailty. Thus, it remains to be seen whether our findings can be transferred to younger AS patient populations with fewer comorbidities and cardiovascular risk factors, such as those who may be eligible for open heart surgery.

Interestingly, we found no association between outcome after TAVI and SDB prevalence in our patients. This in accordance with the lack of association between prevalence, type or severity of SDB with systolic heart function, but stands in contrast to the negative impact of SDB on the prognosis of patients with systolic non-valvular CHF [29]. A plethora of clinical factors, such as kidney function, peripheral arterial disease, chronic obstructive pulmonary disease or frailty, as well as intraprocedural parameters have been described as predictors for the outcome after TAVI [13, 30–32]. In particular, peri-prosthetic aortic regurgitation, which switches the exposition of the left ventricle from pressure load to volume overload, has an important impact on mortality [30]. It seems that amongst these heterogeneous parameters, SDB does not stick out as a single, powerful predictor of outcome. We and others have recently shown, in small patient populations, that the degree of SDB as indicated by AHI significantly improves in patients after successful TAVI but does so almost exclusively in patients with CSA [10, 33]. Future studies must show whether patients with SDB that remains after interventional or surgical valve replacement have a worse prognosis than those patients in whom SDB resolves.

### Limitations

The study was conducted by means of an ambulatory polygraphy system, whereas the ‘gold standard’ for diagnosing SDB is level 1 polysomnography. However, a recent meta-analysis has argued that portable polygraphy has good sensitivity and specificity in diagnosing OSA [34]. Diagnostic accuracy for CSA has been less well investigated. Thus, there might be some degree of inaccuracy in distinguishing between OSA and CSA in our study. The sPAP was estimated only by Doppler echocardiography, the accuracy of which has been questioned [35].

## Conclusions

There is a high prevalence of SDB (71%), with a preponderance of the CSA subtype (46%), in patients with severe aortic stenosis scheduled for TAVI. With increasing severity of SDB as indicated by the AHI, the prevalence of CSA increases at the expense of OSA. This finding is independent of the left ventricular function and is not associated with outcomes after TAVI.
